# *MDM4* genetic variants and risk of gastric cancer in an eastern chinese population

**DOI:** 10.18632/oncotarget.14666

**Published:** 2017-01-14

**Authors:** Meng-Yun Wang, Ming Jia, Jing He, Fei Zhou, Li-Xin Qiu, Meng-Hong Sun, Ya-Jun Yang, Jiu-Cun Wang, Li Jin, Ya-Nong Wang, Qing-Yi Wei

**Affiliations:** ^1^ Cancer Institute, Collaborative Innovation Center for Cancer Medicine, Fudan University Shanghai Cancer Center, Shanghai, China; ^2^ Department of Oncology, Shanghai Medical College, Fudan University, Shanghai, China; ^3^ Department of Medical Oncology, Fudan University Shanghai Cancer Center, Shanghai, China; ^4^ Department of Pathology, Fudan University Shanghai Cancer Center, Shanghai, China; ^5^ Ministry of Education Key Laboratory of Contemporary Anthropology, State Key Laboratory of Genetic Engineering, School of Life Sciences, Fudan University, Shanghai, China; ^6^ Fudan-Taizhou Institute of Health Sciences, Taizhou, Jiangsu, China; ^7^ Department of Abdominal Surgery, Fudan University Shanghai Cancer Center, Shanghai, China; ^8^ Duke Cancer Institute, Duke University Medical Center, Durham, NC, USA

**Keywords:** Case-control study, gastric adenocarcinoma, genetic susceptibility, *MDM4*, polymorphism

## Abstract

MDM4 is a p53-interacting protein and plays an important role in carcinogenesis. In this study of 1,077 gastric cancer (GCa) cases and 1,173 matched cancer-free controls, we investigated associations between three tagging single nucleotide polymorphisms (SNPs) (rs11801299 G>A, rs1380576 C>G and rs10900598 G>T) in *MDM4* and gastric cancer risk in an Eastern Chinese Population. In logistic regression analysis, a significantly decreased GCa risk was associated with the rs1380576 GG variant genotype (adjusted odds ratio [OR] =0.74, 95% confidence interval [CI] =0.56-0.98) under a recessive model, which remained significant after correction by the false-positive reporting probability. This risk was more evident in subgroups of older subjects, males, never smokers, never drinkers and cancers of non-cardia. We then performed SNP-mRNA expression correlation analysis and found that the GG variant genotype was associated with significantly decreased expression of *MDM4* mRNA in normal cell lines for 44 Chinese (*P=*0.032 for GG vs. CC) as well as for 269 multi-ethnic subjects (*P*<0.0001 for GG vs. CC). Our results suggest that the *MDM4* rs1380576 G variant may be markers for GCa susceptibility. Larger, independent studies are warranted to validate our findings.

## INTRODUCTION

Gastric cancer was the fourth most common cancer worldwide and is the second most common cause of death from cancer, with an estimated 951,600 new cases and 723,100 deaths in 2012 [1]. Up to now, the etiology of gastric cancer is still unclear, although multiple factors are thought to play a role in gastric carcinogenesis, including *Helicobacter pylori (H. pylori)* infection [2], nutrition deficiency, high intake of various traditional salt-preserved foods or salt and chemical carcinogenesis existing in tobacco [3, 4]. However, even when exposed to similar exogenous risk factors, only a subset of individuals will develop gastric cancer, suggesting endogenous genetic variation may also contribute to individual susceptibility to gastric cancer.

The p53 pathway has been shown to be crucial in preventing tumor formation, and the disruption of p53 function commonly leads to the initiation or progression of tumors [5]. The murine double minute protein MDM2 is an established regulator of p53, which can directly bind to p53 protein, inhibit its activity and lead to its degradation via the ubiquitination pathway [6]. As a structural homolog of MDM2, MDM4 has recently emerged as another p53-interacting protein, which directly binds to the p53 transactivation domain, inhibits its transcriptional activity, and thus contributes to tumor formation and progression [7]. Additionally, MDM4 can also interact with MDM2 protein via the RING finger domain and inhibit degradation of the MDM2 protein, regulating the role of MDM2 in inhibiting the p53 activity [7, 8].

The central role of MDM4 in regulating p53 activity and human cancer has been highlighted by many studies. For example, mouse knock-out studies showed that the *Mdm4*-knockout mice results in p53-dependent embryonic lethality with defects in proliferation and no apoptosis, which were rescued by knocking out the p53 gene, suggesting the biological role of the p53–MDM2–MDM4 interaction and the major function of these molecules during embryonic development [9–11]. Furthermore, the amplification or over-expression of the human *MDM4* gene has been observed in a large subset of human tumors, including glioma, stomach, soft tissue sarcoma, head and neck squamous carcinoma, retinoblastoma, melanoma, and breast cancer [12–14]. There is also evidence that over-expression of *MDM4* was associated with not only tumor progression but also worse prognosis [13, 15].

Since the p53-MDM4 pathway plays a critical role in response to DNA damage and preventing cancer pathogenesis, we hypothesized that common variants of *MDM4* might be associated with gastric cancer risk. Previous studies have investigated three common tagging SNPs (rs11801299 G>A and rs1380576 C>G in 3′-untranslated region [3′-UTR] and rs10900598 G>T in 5′-UTR) of the *MDM4* gene with risk of oral cancer, squamous cell carcinoma of oropharynx and squamous cell carcinoma of the head and neck and got some positive findings [16–18]. Recently, it was reported that *MDM4* rs1380576 was not associated with gastric cancer risk in a hospital-based Chinese population with a relatively small sample size (642 cases and 720 cancer-free controls) [19]. Here, we reported a relatively large hospital-based case-control study of 1,077 gastric cancer patients and 1,173 cancer-free controls in an Eastern Chinese population to evaluate associations between three common tagging SNPs of *MDM4* and gastric cancer risk. To provide additional mechanistic support for the findings, we performed SNP-mRNA expression correlation analysis to unravel the underlying molecular mechanisms.

## RESULTS

### Characteristics of the study population

The frequency distributions of selected variables between GCa cases and controls are described in Table 1. There was no significant difference in the distributions of age and sex between the cases and the controls (*P =* 0.733 and *P =*0.161, respectively) because of frequency matching. Compared with the cases, the controls were more likely to be smokers and drinkers, but these variables (i.e., age, sex, smoking status and drinking status) were further adjusted for in the subsequent multivariate logistic regression analyses. Of the cases, 295 (27.4%) had gastric cardia adenocarcinoma (GCA), and 782 (72.6%) had gastric non-cardia adenocarcinoma (NGCA).

**Table 1:** Frequency distribution of demographic characteristics of gastric cancer cases and cancer-free controls

Variables	Cases No. (%)	Controls No. (%)	*P* ^a^
All subjects	1,077 (100.0)	1,173 (100.0)	
Age, yr			0.733
Range	21-86	22-86	
Mean^b^	58.6 ±11.30	58.8 ±11.7	
≤ 50	225 (20.9)	259 (22.1)	
51-60	366 (34.0)	378 (32.2)	
61-70	328 (30.4)	371 (31.6)	
>70	158 (14.7)	165 (14.1)	
Sex			0.161
Males	771 (71.6)	808 (68.9)	
Females	306 (28.4)	365 (31.1)	
Smoking status			<0.0001
Never	653 (60.6)	596 (50.8)	
Ever	424 (39.4)	577 (49.2)	
Drinking status			0.015
Yes	258 (24.0)	334 (28.5)	
No	819 (76.0)	839 (71.5)	
Pack-years			<0.0001
0	653 (60.6)	596 (50.8)	
≤ 25 (mean)	220 (20.4)	344 (29.3)	
> 25 (mean)	204 (18.9)	233 (19.9)	
Tumor site			
GCA	295 (27.4)	—	
NGCA	782 (72.6)	—	

GCA, gastric cardia adenocarcinoma; NGCA, non-gastric cardia adenocarcinoma.

^a^ Two-sided χ^2^ test for distributions between cases and controls.

^b^ Data are mean ± SD.

### Associations between *MDM4* genotypes and risk of gastric cancer

**Figure 1:**
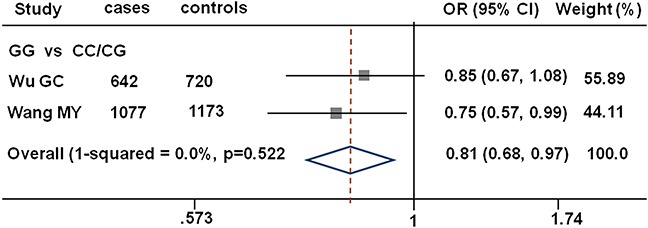
Forest plot showing associations between *MDM4* rs1380576 and gastric cancer risk The ORs and 95% CIs were obtained using GG vs. CC/CG. The axis corresponds to the OR. The diamonds and the horizontal bars represent the overall ORs with 95% CIs given by their width. CI,confidence interval; OR, odds ratio.

The genotype distributions of the three SNPs among the cases and controls and their associations with gastric cancer risk are summarized in Table 2. The genotype frequencies among the controls were in agreement with the Hardy-Weinberg equilibrium (all *P* > 0.05). Compared with the CC/CG genotype carriers under the recessive genetic model, the rs1380576 variant GG genotype carriers had significantly decreased risk of gastric cancer (adjusted OR = 0.74, 95% CI = 0.56–0.98). Finally, we performed a mini-meta analysis of rs1380576 with our and another published study (Figure 1). Consistently, we found that rs1380576 in a recessive model was associated with a significantly decreased risk of gastric cancer (the pooled OR= 0.81; 95% CI = 0.68–0.97 for GG vs. CC/CG) based on 1719 cases and 1893 controls in the pooled analysis.

**Table 2:** Logistic regression analysis of associations between the genotypes of *MDM4* and gastric cancer risk

Variants	Genotypes	Cases(N=1,077)	Controls(N=1,173)	*P* ^a^	Crude OR(95% CI)	*P*	Adjusted OR(95%CI) ^b^	*P* ^b^
rs10900598								
	GG	547 (50.8)	604 (51.5)	0.366	1.00		1.00	
	GT	447 (41.5)	462 (39.4)		1.07 (0.90-1.27)	0.457	1.08 (0.90-1.28)	0.409
	TT	83 (7.7)	107 (9.1)		0.86 (0.63-1.17)	0.326	0.87 (0.64-1.19)	0.379
	GG/GT	994 (92.3)	1066 (90.1)		1.00		1.00	
	TT	83 (7.7)	107 (9.1)		0.83 (0.62-1.12)	0.228	0.84 (0.62-1.14)	0.263
rs11801299								
	GG	380 (35.3)	449 (38.3)	0.657	1.00		1.00	
	GA	539 (50.1)	532 (45.4)		**1.20 (1.00-1.44)**	**0.052**	**1.21 (1.01-1.45)**	**0.043**
	AA	158 (14.7)	192 (16.4)		0.97 (0.76-1.25)	0.827	0.96 (0.75-1.24)	0.770
	GG/GA	919 (85.3)	981 (83.6)		1.00		1.00	
	AA	158 (14.7)	192 (16.4)		0.88 (0.70-1.11)	0.269	1.03 (0.91-1.16)	0.684
rs1380576								
	CC	487 (45.2)	552 (47.1)	0.044	1.00		1.00	
	CG	493 (45.8)	485 (41.4)		1.15 (0.97-1.37)	0.112	1.15 (0.97-1.37)	0.114
	GG	97 (9.0)	136 (11.6)		0.81 (0.61-1.08)	0.147	0.79 (0.59-1.06)	0.117
	CC/CG	980 (91.0)	1037 (88.4)		1.00		1.00	
	GG	97 (9.0)	136 (11.6)		**0.76 (0.57-0.99)**	**0.045**	**0.74 (0.56-0.98)**	**0.034**

CI,confidence interval; OR, odds ratio.

^a^ Chi square test for genotype distributions between cases and controls.

^b^ Adjusted for age, sex, smoking and drinking status in logistic regress models.

The results were in bold, if the 95% CI excluded 1 and *P* < 0.05.

### Stratification and haplotype analysis

In stratification analyses, as shown in Table 3, by assuming a recessive genetic model, we found that the significantly decreased risk associated with rs1380576 GG variant genotype was more evident in subgroups of males (adjusted OR = 0.72, 95% CI = 0.52-0.99), never-smokers (adjusted OR = 0.57, 95% CI = 0.39-0.84) and subjects with non-cardia cancer (adjusted OR = 0.68, 95% CI = 0.50-0.93). Likewise, we found similar results for those carrying “1-2” risk genotypes among subgroups of older subjects (defined as subjects > 59 years old), males, never-smokers, never-drinkers and subjects with non-cardia cancer.

**Table 3:** Stratification analysis for associations between rs1380576 G>C variant genotypes and gastric cancer risk

Variables	Cases/Controls		Crude OR(95% CI)	*P*	Adjusted OR(95% CI)	*P ^a^*	*P_hom_*
	CG/GG	CC					
Age							
≤59	507/525	46/68	0.70 (0.47-1.04)	0.076	0.68 (0.46-1.01)	0.057	0.600
>5	473/512	51/68	0.81 (0.55-1.19)	0.287	0.82 (0.55-1.21)	0.307	
Sex							
Females	283/331	23/34	0.79 (0.46-1.38)	0.406	0.81 (0.46-1.41)	0.454	0.820
Males	697/706	74/102	0.74 (0.54-1.01)	0.057	**0.72 (0.52-0.99)**	**0.044**	
Smoking status							
Never	602/524	51/72	**0.62 (0.42-0.90)**	**0.012**	**0.57 (0.39-0.84)**	**0.005**	0.103
Ever	378/513	46/64	0.98 (0.65-1.46)	0.904	0.99 (0.66-1.48)	0.948	
Drinking status							
Never	745/741	74/98	0.75 (0.55-1.03)	0.078	0.73 (0.53-1.01)	0.055	0.963
Ever	235/296	23/38	0.76 (0.44-1.32)	0.323	0.77 (0.45-1.33)	0.353	
Site							
Cardia	262/1037	33/136	0.96 (0.64-1.44)	0.846	0.89 (0.59-1.34)	0.573	0.184
Non-cardia	718/1037	64/136	**0.68 (0.50-0.93)**	**0.015**	**0.68 (0.50-0.93)**	**0.016**	

CI, confidence interval; OR, odds ratio.

^a^ Obtained in logistic regression models with adjustment for age, sex, smoking status and drinking status.

*P*_hom_ derived from the homogeneity test.

The results were in bold, if the 95% CI excluded 1 or *P* < 0.05.

By using the SAS PROC HAPLOTYPE program, we inferred all the possible haplotypes based on the observed genotype data, of which three common (>10%) haplotypes (GAC, GGG, and TGC) represented 99.4% of all haplotypes for the cases and 97.3% for the controls (Table 4). When the most common haplotype GAC was used as the reference, none of the haplotypes were associated with a significant risk of gastric cancer in logistic regression models, either in a univariate model or a multivariate model with adjustment for age, sex, smoking and alcohol use.

**Table 4:** Haplotype analysis for genotypes of *MDM4* and Gastric Cancer risk

Haplotypes^a^	Haplotype frequencies				Crude OR(95% CI)	*P*	Adjusted OR(95% CI)	*P* ^a^
	Cases		Controls					
	N	%	N	%				
G-A-C	848	48.8	889	51.2	1.00		1.00	
G-G-G	684	48.4	730	51.6	1.01 (0.88-1.16)	0.933	1.01 (0.87-1.16)	0.945
T-G-C	608	48.3	652	51.8	1.00 (0.87-1.16)	0.987	1.01 (0.87-1.17)	0.889

^a^ Obtained in logistic regression models with adjustment for age, sex, smoking status and drinking status. CI, confidence interval; OR, odds ratio.

### Genotype-phenotype correlation analysis

**Figure 2:**
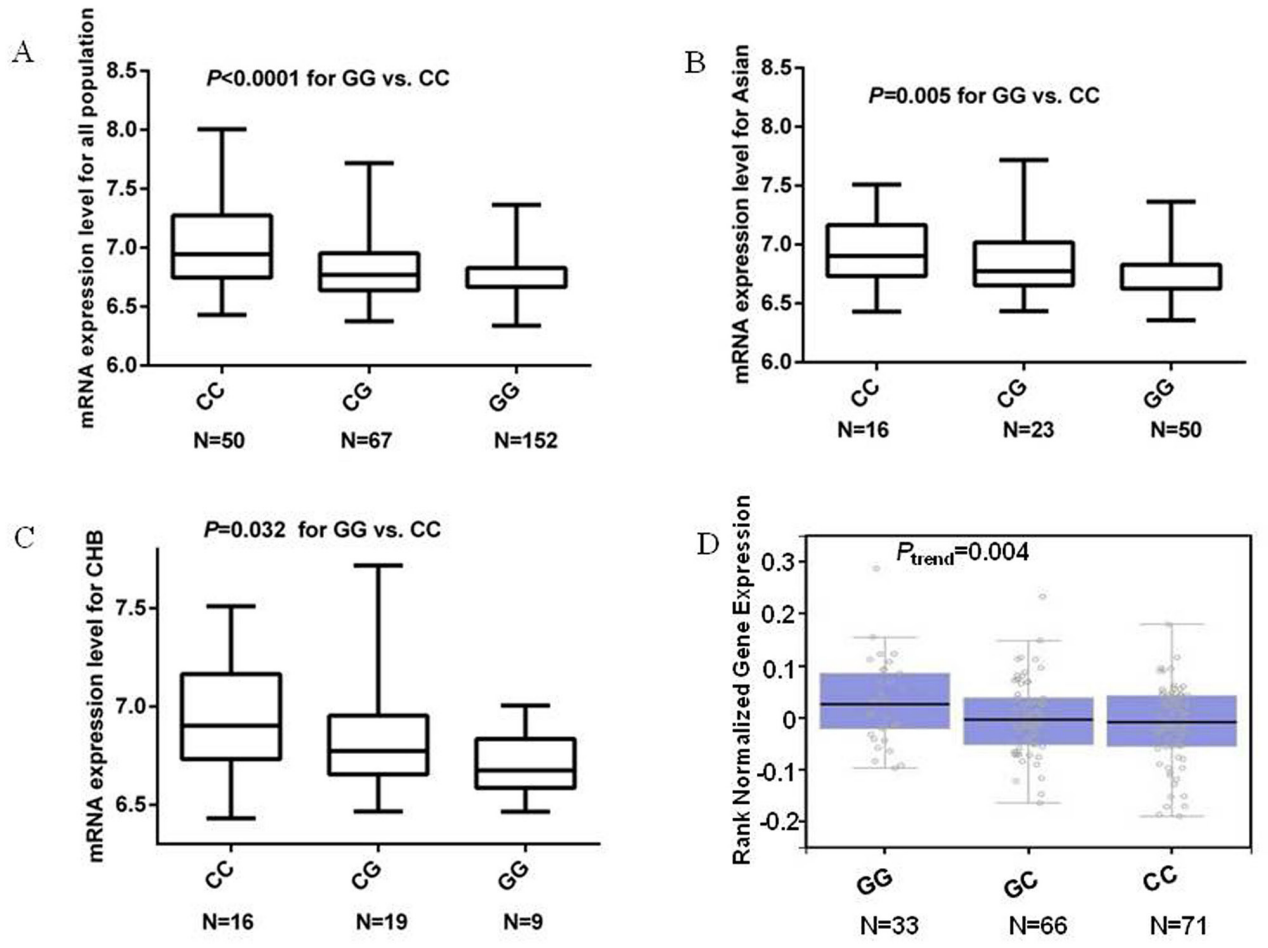
mRNA expression level of the *MDM4* gene in EBV-transformed lymphoblastoid cell lines **A**. mRNA expression in 269 HapMap cell lines. **B**. mRNA expression in 89 HapMap cell lines of unrelated Asian. **C**. mRNA expression in 44 HapMap cell lines of unrelated Han Chinese in Beijing, China. **D**. eQTL results from the GTEx for rs1380576 in stomach tissue.

In addition, we also performed the genotype-phenotype correlation analysis for the *MDM4* rs1380576 C>G SNP by using publically available genotyping data and mRNA expression data of *MDM4* from the 270 lymphoblastoid cell lines. We found that the GG genotype, compared with the CC genotype, appeared to be correlated with significantly decreased mRNA expression, which is consistent for Asians (n = 89, *P* = 0.005), Chinese (n = 44, *P* = 0.032), and all populations (n = 269, *P* <0.0001) (Figure 2). We searched the GTEx database (http://www.gtexportal.org/home) [20] and performed eQTL analysis of rs1380576 for the stomach tissue, and we found that the GG genotype was associated with an increased mRNA expression level of *MDM4*, compared with the CC genotype (Figure 2). However, the eQTL result of the allelic effect in this database was in the different direction from the results from lymphoblastoid cell lines.

## DISCUSSION

We investigated the associations of three tagging SNPs of *MDM4* with risk of gastric cancer in this large, ethnic-specific single institutional case-control study. We found a significant association of the rs1380576 variant GG genotype in *MDM4* with a decreased gastric cancer risk under a recessive genetic model, especially among subgroups of males, never-smokers and subjects with non-cardia cancer. Given the role of *MDM4* in activating gene expression and influencing the p53 activity, it is biologically plausible that the *MDM4* SNPs may modulate risk of gastric cancer.

MDM4, located on chromosome1q32, is a member of the mouse double minute oncoprotein family, which includes the full-length MDM2, MDM4 and their derivate minor forms. MDM4 was initially described as an MDM2 homologue with high similarity with the primary structural level [7, 21]. However, unlike MDM2, MDM4 does not have appreciable ubiquitin ligase activity. Careful mouse genetic studies indicate that MDM4 contributes more to inhibition of p53-mediated transcriptional transactivation while MDM2 contributes more to degradation of p53 [22]. Additionally, it has been shown that through their RING domains, MDM4 binds MDM2 and enhances the ability of MDM2 to regulate p53. Although MDM4 inhibits p53 function, homozygous Myc-tagged MDM4 transgene expression was embryonic lethal, and this could not be rescued with deletion of p53, suggesting a p53-independent function of MDM4 in development. Currently, the development of molecules that block p53-MDM2/MDM4 interactions is considered a promising strategy to combat cancers that contain inactive wild-type p53.

In the present study, the rs1380576 GG variant genotype in *MDM4* was found to be associated with a decreased gastric cancer risk under a recessive genetic model, which only showed some trend but did not reach statistically significance in another Chinese study. The discrepancy may be due to the different population selected. Our patients and controls came from Eastern China, while in another Chinese study, patients and controls came from North of China. The second reason may be due to the different sample size. In the stratification analysis, we found that risk effect of the rs1380576 variant GG genotype was more obvious in subgroups of older subjects, males, never smokers, never drinkers and cancers of non-cardia. This is consistent with the notion that susceptible individuals are likely to have had a light exposure. Never smokers and never drinkers were defined as those who may have exposed to low levels of such exposures as a result of being exposed to passive smoking or other unknown carcinogens in the environment, and, therefore, genetic variation may play a major role in carcinogenesis in such subgroups. Variation by sex may reflect disparate acquisition of risk factors such as *H pylori* infection and Barrett's esophagus [23, 24]. Furthermore, variation by sex or a late age onset of gastric cancer in women compared with that in men may also reflect a protective effect of estrogen in women [25, 26]. Gastric cancer can generally be classified into two categories: cardia gastric cancer (CGC) arising in the area of the stomach adjoining the esophageal-gastric junction, and non-CGC (NCGC) arising from more distal regions of the stomach. Several investigators have suggested that cardia gastric cancer may have distinct risk factors, clinical features and biological behavior compared with non-CGC [22, 27, 28]. Risk factors for CGC include obesity, GORD and Barrett's esophagus, a metaplastic condition that can result from GORD [8, 9, 29, 30]]. NCGC, however, is strongly associated with *H. pylori* infection [14], and the influence of socioeconomic status (SES) also differs. While indicators of low SES such as household crowding, low income, low education and increased number of siblings are positively associated with NCGC, they do not appear to be associated with CGC [16, 17]. In the present study, the combined risk genotypes were more evident in patients with NCGC, which may be due to different biological entities and tumor site-specific etiologies. It may also be due to the relatively small sample size in each subgroup, and, therefore, larger studies with a more stringent design are needed to validate our findings.

To further identify molecular mechanisms underlying our findings, we performed genotype-phenotype correlation analysis of mRNA expression levels for *MDM4* by using publically avaialble genotyping and mRNA expression data. The observed significantly decreased expression of the *MDM4* gene associated with the rs1380576 GG variant genotype may have lead gastric cancer susceptibility in this study population. The eQTL results of the allelic effect from GTEx with stomach tissue were in the different direction from the results of lymphoblastoid cell lines, which may be due to the tissue specificity. Therefore, other studies with larger numbers of gastric tissues are needed to validate our findings. As rs1380576 is a tag SNP, this SNP is in fact in LD with several other potential functional SNPs in *MDM4* that may affect the protein function at the translational level. Thus, fine mapping of this gene will be necessary to identify additional causal variants in the future.

In summary, the present study investigated the associations between three *MDM4* tagging SNPs and gastric cancer risk with a relatively large sample size. Several limitations of our study need to be addressed. Firstly, this hospital-based case-control study may have selection bias and information bias, which may be minimized by frequency-matching cases and controls as well as the adjustment for potential confounding factors in the final analyses. Second, only three tagging SNPs of *MDM4* were investigated in the present study, which did not cover all SNPs and may have missed some important variants within *MDM4*. Third, other risk factors, especially the *H. pylori* infection status, were not available for further analysis due to the nature of the retrospective study design. This limitation should be overcome in our future studies. Fourth, we were not able to measure the expression of *MDM4* in mRNA and protein levels due to the lack of clinic tissues/samples. Finally, although our sample size was relatively large, the small sample size in subgroup analyses may have limited statistical power. Hence, our findings need to be confirmed by studies with larger sample sizes. Despite these limitations, our findings are biologically plausible and provide some novel clues for the role of *MDM4* in the development of gastric cancer.

## MATERIALS AND METHODS

### Study population

Patients with newly diagnosed, histopathologically confirmed, and untreated primary gastric adenocarcinoma were recruited between January 2009 and March 2011 at Fudan University Shanghai Cancer Center. All patients came from Eastern China, including Shanghai City, Jiangsu Province, Zhejiang Province and the surrounding regions. Participants who had gastric adenosquamous, squamous cell carcinoma, neuroendocrine tumor, stromal tumor, metastasized cancer from other organs or any histopathologic diagnosis other than gastric adenocarcinoma were excluded from this study. Cancer-free controls were recruited from a large prospective cohort recruited for Taizhou longitudinal study (TZL) at the same time period in the Eastern China with the selection criteria including no individual history of cancer. These cancer-free Han Chinese controls were frequency matched to cases on age (± 5 years) and sex [31]. A structured questionnaire was used to obtain the following information from each of the participants during personal interviews: age, sex, ethnicity, smoking and alcohol consumption. After interview, each participant donated a sample of approximately 10-mL blood, of which 1 mL was used for genomic DNA extraction. This research protocol was approved by the Institutional Review Board of FUSCC and the experiment on humans was performed in accordance with relevant guidelines and regulations.

### SNP Genotyping

Genomic DNA extraction and genotyping were conducted as described previously [32], with a successful genotyping rate of 99.5% by using the TaqMan probe assays (Applied Biosystems, Foster City, CA, USA) on a 7900 HT sequence detector system (Applied Biosystems) according to the manufacturer's protocol. More than 10% of the samples were retested for each polymorphism randomly, and the results were 100% concordant.

### Genotype and mRNA expression data of lymphoblastoid cell lines from hapmap database

To explore the functionality of the *MDM4* rs1380576 C>G SNP, we used publically available data on genotypes and transcript expression levels of *MDM4* from 270 lymphoblastoid cell lines from all populations (45 Han Chinese in Beijing, China +45 Japanese in Tokyo, Japan +90 Utah residents with Northern and Western European ancestry +90 Yoruba in Ibadan, Nigeria) available online for the genotype–phenotype correlation analysis [33]. The genotyping data were from the HapMap phase II release 23 data set consisting of 3.96 million SNP genotypes from these 270 individuals.

### Statistical analysis

Differences in the distributions of demographic characteristics, selected variables, and frequencies of genotypes between cases and controls were tested by the Student's *t*-test (for continuous variables) or Chi-square-test (for categorical variables). Hardy-Weinberg equilibrium (HWE) was evaluated by a goodness-of-fit Chi-square test to compare the observed genotype frequencies with the expected among the controls. Univariate and multivariate logistic regression models were used to evaluate associations between genotypes and risk of gastric cancer by odds ratios (ORs) and their 95% confidence intervals (CIs) with the adjustment for possible confounders. Risk genotypes of studied SNPs were combined to create a genetic score of the number of the observed risk genotypes, and this score was used for further analyses. All statistical analyses were performed by using Statistical Analysis Software (v.9.3 SAS Institute, Cary, NC), and all *P* values were two-sided with a 0.05 significance level.

## Abbreviations

SNPs: single nucleotide polymorphisms
GCa: gastric cancer
OR: odds ratio
CI: confidence interval
MDM4: murine double minute protein4
HWE: Hardy-Weinberg equilibrium
GCA: gastric cardia adenocarcinoma
NGCA: non-cardia adenocarcinoma.
